# An Examination of Training Quality and Provider Outcomes Across Two Generations of Train-the-Trainer

**DOI:** 10.1007/s10488-025-01463-w

**Published:** 2025-08-21

**Authors:** Catherine A. Callaway, Joshua M. Varghese, Emma R. Agnew, Laurel D. Sarfan, Allison G. Harvey

**Affiliations:** 1 Department of Psychology, University of California, Berkeley, 2121 Berkeley Way, Berkeley, CA 94720 USA; 2 Department of Research and Evaluation, Kaiser Permanente Southern California, 100 S. Los Robles, Pasadena, CA 91101 USA

**Keywords:** Train-the-trainer, Implementation, Sustainment, Community mental health, Transdiagnostic, Serious Mental Illness

## Abstract

**Abstract:**

Train-the-trainer (TTT) is a promising method for implementing and sustaining evidence-based psychological treatments (EBPTs) in routine practice. In TTT, external “expert” trainers train an initial provider cohort (i.e., Generation 1) and then train “local” trainers to train the next provider cohort (i.e., Generation 2). This study evaluated whether training quality and provider outcomes are maintained across two generations of TTT in a hybrid type 2 effectiveness-implementation trial. TTT was used to implement the Transdiagnostic Intervention for Sleep and Circadian Dysfunction (TranS-C) in community mental health centers (CMHCs) across California, United States. CMHCs were randomized to receive training in either Standard or Adapted TranS-C. Data from both conditions were combined to maximize power. Two expert trainers trained 115 providers (Generation 1), and 25 local trainers trained 42 providers (Generation 2). Trainings were coded for gold standard training elements (TranS-C content, training techniques) using the Gold Standard Training Checklist. Providers completed a post-training assessment which measured TranS-C knowledge, acceptability, appropriateness, and feasibility of TranS-C, and willingness and confidence to use TranS-C. Local trainers delivered more gold standard training techniques than expert trainers. No differences were found between generations on provider outcomes after correcting for multiple comparisons. Sensitivity analyses excluding outliers revealed that providers trained in Generation 2 rated TranS-C as more appropriate to their setting. The extent of gold standard elements delivered in trainings were not related to provider outcomes. These findings support the potential of TTT to sustain, and even improve, training quality and provider outcomes in a CMHC setting.

**Trial Registration:**

The parent study was registered on ClinicalTrials.gov (NCT04154631) on 11 April 2019, https//clinicaltrials.gov/study/NCT04154631. This study was preregistered on the Open Science Framework (osfregistrationsx7v38v1).

**Supplementary Information:**

The online version contains supplementary material available at 10.1007/s10488-025-01463-w.

## Introduction

Barriers to the implementation of evidence-based psychological treatments (EBPTs) in routine practice settings are complex and exist across individual, organizational, and system levels (Damschroder et al., 2009). Provider training in EBPTs is one such barrier that has been identified by stakeholder groups as both considerable and modifiable (Aarons et al., 2009). Though training alone is not sufficient for EBPT implementation (Beidas & Kendall, 2010; Herschell et al., 2010; Valenstein-Mah et al., 2020), it is a crucial step in the early adoption and long-term sustainability of EBPTs (Stirman et al., 2012).

Community mental health centers (CMHCs) are a care setting that typifies the challenges of EBPT implementation and sustainment. CMHCs are major, publicly funded providers of mental health treatment that serve socioeconomically vulnerable populations (Kilbourne et al., 2018; Lee et al., 2024). Overall penetration rates of EBPTs are low in CMHCs (1−3%; Bruns et al., 2016), which has serious implications given patients have few alternatives if treatments are not effective (Aarons et al., 2009). Thus, implementation research in CMHCs is vitally important to support providers in delivering EBPTs and, in turn, improve outcomes and health equity (Lee et al., 2024).

The past two decades have evidenced many successful efforts to train providers to deliver EBPTs in community settings, with significant improvements in provider knowledge, attitudes, and behaviors post-training (Beidas & Kendall, 2010; Valenstein-Mah et al., 2020). Reviews of clinical training approaches (e.g., Beidas & Kendall, 2010) conclude that the “gold standard” for EBPT trainings include active, experiential learning components, supported by ongoing supervision and consultation. Active learning is an interactive process that uses action and reflection (e.g., modeling, role-plays, feedback) and has been employed successfully in clinical training initiatives (Cross et al., 2007; Sanders & Turner, 2005). Typically, these gold standard training activities are delivered by a small number of program developers or affiliated experts (Triplett et al., 2020). While this approach can help ensure fidelity to the EBPT, reliance on a limited pool of expert trainers severely constrains the scalability and long-term sustainability of training efforts (Massatti et al., 2008; Swain et al., 2010). This challenge is particularly pronounced in CMHCs which often experience high rates of staff turnover and lack the financial resources to support ongoing expert-led training cycles.

The train-the-trainer (TTT) model holds potential to address these barriers and produce effective, sustainable training programs for EBPTs (Frank et al., 2020). In TTT, external expert trainers train an initial cohort of providers in an EBPT. Following prior literature (e.g., Callaway et al., 2023), we will refer to these as “Generation 1 trainings” and the providers trained as “Generation 1 providers.” Next, Generation 1 providers are offered the opportunity to become “local trainers” and receive additional training in how to train others in the EBPT. Local trainers then train the next cohort of providers within their organization. We refer to these as “Generation 2 trainings” and the providers trained as “Generation 2 providers.” TTT is theorized to decrease long-term costs relative to repeated use of an external expert trainer, address the high turnover rate of trained staff, and foster an organizational climate to sustain the EBPT (Brabson et al., 2021). TTT has been used for a variety of populations (e.g., individuals at risk for eating disorders, post-traumatic stress disorder, intellectual disabilities) with promising results (Andzik & Cannella-Malone, 2017; Greif et al., 2015; Smith et al., 2017). Importantly, there is an early signal that TTT appears to confer advantages over other training methods, including in the sustainment of EBPT delivery (Barnett et al., 2021; Fitzsimmons-Craft et al., 2021; Herschell et al., 2021).

The TTT model rests on a critical core assumption: that there is no significant reduction, or “scale-up penalty,” in outcomes across generations of training. As once put by Dr. John Weisz and colleagues, the key question to be tested is: “when the torch is passed, does the flame still burn?” (Weisz et al., 2018). The “transfer of learning” problem from cognitive psychology may offer a helpful theoretical guide to the study of this issue. The transfer of learning problem is the tendency for learned knowledge to remain context-bound, resulting in poor application when the learning and application environments differ (e.g., Leberman et al., 2006). Applied here, the odds may be stacked against a successful transfer of knowledge from expert trainers to local trainers, despite best efforts from both parties. Indeed, for prospective local trainers, the initial learning environment (attending an EBPT training as a trainee) and the application environment (leading an EBPT training as a trainer) differ starkly. Although additional instruction is often provided to prospective local trainers in how to lead an EBPT training to facilitate transfer (see ‘Train the Trainer Process’ for more detail on this study’s procedures), numerous dissimilar elements remain between the ‘learning’ and ‘application’ environments (e.g., audience/trainees present, support available, time pressures). While there may be crucial advantages for local trainers in the trainer role, as they can enrich trainings with context-specific knowledge and garner more trust with trainees (Rogers, 2010; Stice et al., 2023), on balance it seems that the barriers to an effective transfer of knowledge may eclipse these benefits. If the transfer of knowledge from Generation 1 to Generation 2 trainers is unsuccessful, Generation 2 may suffer poorer outcomes, even in the hands of highly motivated and capable local trainers.

As highlighted elsewhere (Callaway et al., 2023), the few TTT studies targeting this line of questioning have produced mixed results. Promisingly, some studies found no difference between generations on training effectiveness (Triplett et al., 2020), provider competence (Wilfley et al., 2020), and patient outcomes (Shore et al., 1995). Unfortunately, there is also evidence of poorer outcomes resulting from trainings led by local trainers (Generation 2) compared to trainings led by expert trainers (Generation 1). Martino et al. (2011) reported that trainees rated local trainers as less skillful, defined as less extensively and skillfully covering EBPT information in a training workshop and follow-up supervision. Southam-Gerow et al. (2014) found that trainees trained by local trainers submitted poorer quality case materials compared to trainees trained by expert trainers, although the case materials were in the proficient range for both groups. In a recent study by Brabson and colleagues (2021), trainees trained by local trainers evidenced less knowledge of the EBPT and were less satisfied with the training, which was hypothesized to be due to local trainers delivering fewer training strategies (e.g., live observation, review of recorded sessions, co-therapy, individual supervision). As TTT becomes more widely used (Frank et al., 2020), more research is needed to ensure key outcomes are maintained after the “torch is passed” from expert to local trainers.

The goal of the present study is to progress knowledge in this domain with a focus on training quality as, to the best of our knowledge, it has yet to be investigated across generations of TTT. This is important as maintaining training quality may be necessary in protecting against program drift and maintaining provider fidelity over time (Cross et al., 2014). This study was conducted within a randomized trial (henceforth “parent study”) that was designed to evaluate the implementation and effectiveness outcomes of a transdiagnostic EBPT for sleep and circadian dysfunction—Transdiagnostic Intervention for Sleep and Circadian Dysfunction (TranS-C)—for serious mental illness (SMI) in community mental health centers (CMHCs). This is the first study to utilize a TTT approach for (a) sleep treatment, (b) adults with SMI, and (c) CMHCs and builds upon recent efforts to sustain transdiagnostic, modular treatments in real-world settings (Weisz et al., 2018). Following the National Institute of Health Stage Model (Onken et al., 2014), this trial was conducted over the course of three phases. In the first phase, the Implementation Phase, treatment experts trained the first cohort of CMHC providers in TranS-C (i.e., Generation 1 of TTT; Sarfan et al., 2023). In the second phase, the Train-the-Trainer Phase, CMHC providers learned to train and supervise their peers in the delivery of TranS-C (i.e., Generation 2 of TTT; Callaway et al., 2023). In the third phase, the Sustainment Phase, sustainment outcomes of TTT were examined (Sarfan et al., 2024). Data for the present study were drawn from the first two phases.

This study has three aims. The first is to compare Generation 1 and Generation 2 trainings on the extent of gold standard TranS-C content covered and the extent of gold standard training techniques used. We hypothesized that, given the transfer of learning problem, Generation 1 trainings led by expert trainers will cover more content and utilize more training techniques relative to Generation 2 trainings led by local trainers. The second aim is to compare Generation 1 and Generation 2 on a post-training assessment, which measures three domains: (a) provider knowledge of TranS-C, (b) provider perceptions of the acceptability, appropriateness, and feasibility of TranS-C, and (c) provider willingness and confidence to use TranS-C. We will refer to these variables as “provider outcomes.” We hypothesized that, given the transfer of learning problem, Generation 1 providers will perform better on these outcomes relative to Generation 2 providers. The third aim is to evaluate the effect of the extent of gold standard TranS-C content covered and the extent of gold standard training techniques used on provider outcomes. We hypothesized that both gold standard elements will be associated with better provider outcomes.

## Methods

### Study Overview

Data for the present study were drawn from a hybrid type 2 effectiveness-implementation trial (R01MH120147), which was designed to evaluate TranS-C in CMHCs across California in the United States. Two variations of TranS-C are tested—Standard TranS-C and Adapted TranS-C—alongside usual care offered by each CMHC. Standard TranS-C is delivered in 8 × 50 min weekly sessions and comprised of four cross-cutting modules featured in every session, four core modules, and seven optional modules used based on clinical presentation (Harvey & Buysse, 2017). In response to the initial efficacy trial of TranS-C in CMHCs (Harvey et al., 2021), Adapted TranS-C was developed to improve fit with the CMHC context. Adapted TranS-C is delivered in 4 × 20 min weekly sessions and has the following modifications: (a) cross-cutting modules are standardized and scripted to reduce preparation time, (b) four core modules are split into five, and (c) one optional module is included. CMHCs were randomized to receive training in either Standard or Adapted TranS-C. CMHCs and their providers were blinded to treatment condition. For the present analyses, no differences were expected between conditions as only shared TranS-C content was included in analyses (see ‘Data Analysis’) and previous research has not shown a relationship between condition and provider perceptions of treatment fit (e.g., Harvey et al., 2025). Thus, data were combined across conditions to maximize power. Treatment condition was tested as a predictor for relevant outcomes and considered as a covariate (see ‘Substantive Analyses’).

For the present study, data collection was stopped on 9/28/2023 due to decreasing study resources available to collect and code training recordings.

### Participants

The participants included in the present analyses are 157 providers (115 Generation 1; 42 Generation 2) and 27 trainers (two Generation 1 expert trainers; 25 Generation 2 local trainers). See Table 1 for demographic characteristics.

**Table 1:** Provider and trainer demographics

Characteristic	Providers (*N* = 157)		Trainers (*N* = 27)	
	Generation 1 (*n* = 115)	Generation 2 (*n* = 42)	Generation 1 (*n* = 2)	Generation 2 (*n* = 25)
	Mean or N (SD or %)	Mean or N (SD or %)	Mean or N (SD or %)	Mean or N (SD or %)
Age (years)	40.81 (11.01)	39.51 (10.05)	39.5 (16.26)	44.53 (9.35)
Sex				
Male	12 (10.43)	2 (4.76)	–	2 (8.0)
Female	101 (87.83)	40 (95.24)	2 (100)	17 (68.0)
Non-binary	–	–	–	–
Prefer not to say	2 (1.74)	–	–	–
Gender identity				
Female/Woman	95 (82.61)	36 (85.71)	2 (100)	16 (64.0)
Male/Man	10 (8.70)	2 (4.76)	–	2 (8.0)
Non-binary/Gender queer	2 (1.74)	1 (2.38)	–	–
Not specified	8 (6.96)	3 (7.14)	–	1 (4.0)
Race				
American Indian /Alaskan Native	4 (3.48)	–	–	–
Black or African American	5 (4.35)	3 (7.14)	–	1 (4.0)
Native Hawaiian or Pacific Islander	1 (0.87)	–	–	–
Asian	15 (13.04)	8 (19.05)	–	2 (8.0)
White	71 (61.74)	27 (64.29)	2 (100)	14 (56.0)
More than one race	8 (6.96)	2 (4.76)	–	1 (4.0)
Not specified	11 (9.57)	2 (4.76)	–	1 (4.0)
Ethnicity				
Hispanic or Latino	38 (33.04)	10 (23.81)	–	4 (16.0)
Not Hispanic or Latino	64 (55.65)	30 (71.43)	2 (100)	13 (52.0)
Not specified	13 (11.3)	2 (4.76)	–	2 (8.0)
Employment (years)	3.93 (4.05)	4.74 (5.87)	7.5 (10.60)	6 (5.12)
Total Experience in Role^a^ (years)	7.53 (6.99)	7.54 (8.78)	12 (9.89)	11.58 (7.17)
Caseload Number	35.33 (32.59)	34.41 (39.13)	–	38.77 (53.53)
Degree^b^				
Marriage and family therapy	35 (30.43)	4	–	3 (12.0)
Psychology	15 (13.04)	6	1 (50)	5 (20.0)
Social work	40 (34.78)	19	1 (50)	4 (16.0)
Nursing	1 (0.87)	8	–	1 (4.0)
Medical	3 (2.61)	1	–	–
Occupational Therapy	6 (5.22)	–	–	2 (8.0)
Other	11 (9.57)	5	–	1 (4.0)
Theoretical Orientation^b^				
Client-centered	84 (73.04)	37 (88.10)	1 (50)	11 (44.0)
Family systems	30 (26.09)	8 (19.05)	–	1 (4.0)
Cognitive Behavioral Therapy	72 (62.61)	23 (54.76)	2 (100)	13 (52.0)
Psychodynamic	28 (24.35)	12 (28.57)	–	3 (12.0)
Humanistic	4 (3.48)	9 (21.43)	–	1 (4.0)
Integrative/Holistic	4 (3.48)	2 (4.76)	–	2 (8.0)
None	4 (3.48)	–	–	–
Licensed	71 (61.74)	28 (66.67)	2 (100)	14 (56.0)
Received Previous Training in Sleep Problems	9 (7.83)	2 (4.76)	2 (100)	5 (20.0)

Demographic information for Generation 2 trainers was pulled from the post-training assessment the trainer completed as a provider. Demographic information is missing from six Generation 2 local trainers

^a^Total Experience in Role = Employment (years in current role) plus the number of years working in a similar role with a different employer

^b^Participants could select multiple response options

Across both generations, each CMHC determined which providers were eligible to receive TranS-C training within their site (e.g., case managers, nurses, psychiatrists). Eligible local trainers had completed a Generation 1 training and (a) were employed or able to deliver patient-facing services to CMHC patients, (b) were interested in learning and delivering TranS-C, and (c) volunteered to participate. Most local trainers had completed their TranS-C certification, including completing TranS-C with three patients (see Sarfan et al., 2023 for more details), or were actively delivering TranS-C to patients and progressing towards TranS-C certification. The UC Berkeley facilitation team worked collaboratively with CMHC leadership and CMHC champions (providers actively engaged in and spearheading the TranS-C program) to identify and approach potential local trainers for participation. Participants were consented prior to participation and informed that they could withdraw from the study at any time.

Providers completed an assessment after TranS-C trainings (henceforth “post-training assessment”). For in-person trainings, providers could complete a hard-copy post-training assessment and data were entered by the UC Berkeley team. For trainings held online (the vast majority; see ‘Train-the-Trainer Process’), providers completed the assessment via a secure Qualtrics link. Each training was video recorded. Recordings were handled and securely stored by the UC Berkeley team. The University of California, Berkeley, Committee for the Protection of Human Subjects approved all eligibility criteria and study procedures (2019-04-12091), which are described in greater detail elsewhere (Callaway et al., 2023; Sarfan et al., 2023). Consent was obtained by all participants.

### Train-the-Trainer Process

#### Generation 1 Trainings

UC Berkeley facilitators organized regular Generation 1 TranS-C trainings. Standard TranS-C trainings consisted of one, 6-hour workshop or two, 3-hour workshops, based on CMHC preferences. Adapted TranS-C trainings consisted of four, 1-hour or two, 2-hour workshops.

Generation 1 trainings were led by one of two expert trainers: the lead facilitator (licensed clinical social worker), or the treatment developer (licensed clinical psychologist). Of 49 Generation 1 trainings, 32 training recordings were of sufficient length (i.e., at least half of the training successfully recorded) to be coded. All but two Generation 1 trainings were held via Zoom due to COVID-19. Of 246 providers trained in Generation 1, 115 were retained for analyses as they completed the post-training assessment and consented to the study. Of note, nine of the 115 providers included here were excluded from the parent study (e.g., Harvey et al., 2025), as the parent study required each provider to have treated at least one patient to be designated as a Generation 1 provider. These nine providers are included in the present sample because although they did not treat a patient with TranS-C within the larger study, they met the criteria listed above (completed post-training assessment and consented to the study).

#### Training Local Trainers

In preparation for Generation 2, expert trainers trained local trainers to deliver TranS-C trainings. This training was developed to match the time-constrained CMHC context. As discussed elsewhere (see Callaway et al., 2023 for more detail), the expert trainer led a 30–60 min welcome meeting which provided an overview of the training process and tips for public speaking (e.g., practicing aloud, speaking at a deliberate pace, navigating unexpected questions from trainees). Next, the expert trainer conducted TranS-C “booster” trainings which reviewed the training content in one-hour chunks (seven hours for Standard, five hours for Adapted). Additionally, the expert trainer offered 30–60 min 1-on-1 consultations with local trainers to answer questions and provide additional practice time. To facilitate the transfer of learning from the practice to application contexts, each local trainer was assigned a selection of slides to present in booster trainings ‘live’ to their colleagues and the expert trainer. To assist in their preparation, they were also provided a video recording of the expert trainer presenting the material. Local trainers were deemed ready to lead their first training after actively participating in and completing all booster trainings.

#### Generation 2 Trainings

Local trainers led Generation 2 trainings independently. Like Generation 1 trainings, Standard TranS-C trainings consisted of one, 6-hour workshop or two, 3-hour workshops, and Adapted TranS-C trainings consisted of four, 1-hour or two, 2-hour workshops, based on CMHC preferences.

For their first training, a UC Berkeley facilitator attended to provide technological support and answer questions if invited. For subsequent trainings, facilitator support was offered but not required. Of 29 Generation 2 trainings, 24 training recordings were of sufficient length (i.e., at least half of the training was successfully recorded) to be coded. All Generation 2 trainings were held via Zoom. Of 110 providers trained in Generation 2, 42 were retained for analyses as they completed the post-training assessment and consented to the study. One of the 42 providers included here was excluded from the parent study (e.g., Harvey et al., submitted) for the same rationale described above.

### Measures

#### Gold Standard Training Checklist

The Gold Standard Training Checklist was developed for this study. It captures both training content and technique items, in line with similar measures developed for clinical supervision (Dorsey et al., 2018; Garland et al., 2010). The checklist includes detailed descriptions of and examples for each item, with additional guidance for item discrimination (see Online Resource 1 for the complete checklist, scoring and coder instructions). This measure was developed and iteratively refined by the treatment developer, an advanced graduate student, and two post-baccalaureate students.

**Content items.** The TranS-C manual (Harvey & Buysse, 2017) was the source for gold standard TranS-C content. A graduate student and two post-baccalaureate students independently reviewed the manual to identify key treatment points, defined as “key content and skills taught during training that the trainer thinks are important for the sleep coach to understand to provide effective sleep coaching to patients.” Key treatment points were refined via group discussion and consultation with the treatment developer. For ease of coding, treatment points were divided into 18 sections, corresponding to 16 TranS-C modules and two additional sections (Overview of TranS-C, Sleep Diary; see Table 2 and Online Resource 1). Sections had an average of 10.5 treatment points (max 27; min 2) for a total of 189 points. Coders calculated the percentage of treatment points that were covered in each section and converted it to a content extensiveness rating, ranging from 0 (not covered) to 5 (extensively covered). For the present analyses, extensiveness ratings were averaged across sections to assign one overall content extensiveness rating for each training. Coders also noted any training material that deviated from gold standard TranS-C content (e.g., “use lavender spray for sleep”) and reported recording mishaps. If a recording mishap compromised the scoring of a content section, this was marked as missing data (see missing data procedures below in ‘Data Analysis’).

**Table 2:** Gold standard training checklist items

Content items	Extensiveness rating (0–5)
Overview of TranS-C^a^ *(16 points)*	
Sleep Diary^a^ *(17 points)*	
*Cross-Cutting Modules*	
Case Formulation^a^ *(14 points)*	
Behavior Change and Motivation^a^ *(15 points)*	
Goal Setting^a^ *(6 points)*	
*Core Modules*	
Establishing Regular Sleep-Wake Times^a^ *(27 points)*	
Learning a Wind-down Routine^a^ *(4 points)*	
Learning a Wake-up Routine^a^ *(11 points)*	
Improving Daytime Functioning^a^ *(11 points)*	
Correcting Unhelpful Sleep-related Beliefs^b^ *(8 points)*	
Maintenance of Behavior Change^a^ *(2 points)*	
*Optional Modules*	
Improving Sleep Efficiency *(7 points)*	
Reducing Time in Bed *(4 points)*	
Dealing with Delayed or Advanced Phase *(11 points)*	
Reducing Sleep-related Worry and Vigilance^a^ *(12 points)*	
Promoting Compliance with CPAP Machine/Exposure Therapy for Claustrophobic Reactions *(7 points)*	
Negotiating Sleep in a Complicated Environment *(12 points)*	
Reducing Nightmares *(5 points)*	
Technique Items	Extensiveness Rating (0–3)
Agenda-Setting * Trainer sets an agenda to set an organizational structure and* *facilitate effective time management.*	
Behavioral Rehearsal (Role-play) * Trainer guides trainees through practicing effective use of a* *therapeutic skill or technique; trainees actively practice a skill* *playing the role of the provider.*	
Trainer Modeling * Trainer models (i.e.*,* enacts or demonstrates) a specific clinical skill or* *method of delivering a treatment component.*	
Socratic Questioning * Trainer uses questions to promote critical thinking and active learning* *among trainees (e.g.*,* probe assumptions*,* reasons*,* evidence)*,* as opposed* *to providing “answers.”*	
Positive Reinforcement/Praise * Trainer searches for*,* identifies*,* and labels positive aspects of* *trainees’ work to reinforce strengths in their TranS-C delivery and* *participation in training.*	
Discussion to Promote Self-reflection * Trainer encourages trainees to explore any thoughts*,* emotions*,* and* *actions that have arisen during the workshop with the goal of* *developing new understandings and appreciations.*	
Critical Collaborative Inquiry * Trainer promotes peer learning by encouraging trainee contributions of* *experiences*,* tips and skills.*	
Other Activities to Promote Active Learning * Trainer uses activities to promote engagement with TranS-C material* *(e.g.*,* conducting polls*,* completing patient worksheets*,* calculating sleep* *variables*,* facilitating group discussion).*	
Trainee Response and Engagement * Trainees engage in discussion and are responsive to trainers’ attempts* *to promote active learning.*	

The Sleep and Circadian Education cross-cutting module of TranS-C was subsumed into the content of the core and optional modules for ease of coding. ‘Points’ refer to key treatment points, defined as: “the key content and skills taught during training that the trainer thinks are important for the sleep coach to understand to provide effective sleep coaching to patients”

^a^Content items included in the present analyses, given they were shared between Standard and Adapted TranS-C (see ‘Data Analysis’ for more details)

^b^This is a standalone core module in Standard TranS-C, whereas it is integrated across all modules in Adapted TranS-C

**Technique items.** Eight gold standard training techniques (see Table 2 and Online Resource 1) were derived via a review of the training literature, including coding manuals in related areas (e.g., supervision, consultation; Dorsey et al., 2018; Nakamura et al., 2014), by a graduate student and two post-baccalaureate students. Seven techniques (agenda-setting, behavioral rehearsal or role-play, trainer modeling, Socratic questioning, self-reflection, critical collaborative inquiry, other activities to promote active learning) invite trainees to engage cognitively and meaningfully with the material, defined as “active learning” (Bonwell & Eison, 1991). These were selected given the mounting evidence that active learning strategies are necessary to influence behavior change post-training (Frank et al., 2020). The final item captures trainee response to and engagement in the training.

Coders assigned a technique extensiveness rating for each item, ranging from 0 (not used) to 3 (extensively used). We considered applying a 0–5 extensiveness scale to training techniques to capture the same range of variability as training content (see ‘Content Items’ above). However, during coding scheme development, it became evident that it was not feasible to establish sufficient anchors across the full 0–5 scale to support reliable coding for training techniques. This was partly due to the heterogeneity in length of training sessions. For example, we would expect more total instances of Socratic questioning in a six-hour Standard TranS-C training compared to a four-hour Adapted TranS-C training. Given that there is little guidance in the existing literature as to the optimal ratio of gold-standard techniques relative to session length, it was difficult to designate from the outset how many instances of Socratic questioning warranted a high score. Thus, we adopted a more constrained 0–3 scale for technique items and provided more nuanced guidance for coders (see complete coder instructions in Online Resource 1) to enhance reliability.

For the present analyses, extensiveness ratings were averaged across techniques to assign one overall technique extensiveness rating for each training. Coders also recorded a raw count of role plays, instances of trainer modeling, and number of breakouts for group discussion to aid in arriving at consensus for the behavioral rehearsal, trainer modeling, and critical collaborative inquiry extensiveness ratings, respectively.

**Scoring and Interrater Reliability.** A consensus scoring approach was selected to maximize reliability of the data and allow for ongoing improvement of the coding manual (Richards & Hemphill, 2018). Each training was scored by three to four undergraduate students. An advanced graduate student, post-bac student, or trained undergraduate student facilitated consensus discussions among coders. Coders were blind to treatment condition (Standard vs. Adapted TranS-C) and generation (1 vs. 2) of trainings. Individuals facilitating consensus discussions did not view nor code the videos, as blinding was not possible when handling data. The coding team completed an intensive training on the Gold Standard Training Checklist led by an advanced graduate student. Interrater reliability was excellent according to two-way mixed effects, consistency, single-rater intraclass correlations (ICC) for three coders (*n* = 1160, ICC = 0.93, *p* < 0.001) and four coders (*n* = 540, ICC = 0.875, *p* < 0.001) (McGraw & Wong, 1996).

#### Provider Outcomes

In addition to the measures below, a sociodemographic form was completed by providers (see Table 1). For these analyses, provider theoretical orientation was classified as ‘CBT’ or ‘non-CBT’, with the latter category combining all other response options (Garcia et al., 2020; Nelson & Steele, 2008), and previous training in sleep treatment was coded as ‘yes’ or ‘no.’

**Knowledge.** A knowledge test comprised of five short-answer questions was developed by the UC Berkeley team, led by the treatment developer. Face validity was confirmed by experienced TranS-C providers to assess knowledge of foundational concepts to TranS-C (e.g., “Why are regular bed and wake times an important aspect of sleep health?”). A short-answer format was selected as it aids in knowledge acquisition (Pyc & Rawson, 2009). Each response was scored as 0 (blank, inaccurate), 1 (correct and/or relevant, does not include required word/phrase/idea), or 2 (correct, includes required word/phrase/idea) for a total score ranging from 0 to 10. The coding team was an advanced graduate student and two experienced research assistants. The training period consisted of independent coding and discussion until the group evidenced “excellent” interrater reliability, defined as an ICC between 0.75 and 1.00 (Cicchetti, 1994). Interrater reliability during the training period was excellent according to a two-way mixed effects, consistency, average ICC (*n* = 44, ICC = 0.99, *p* < 0.001) (McGraw & Wong, 1996). After the training period, knowledge tests were randomly allocated to coders for independent scoring. For knowledge tests coded by more than one team member, a consensus decision was used as final data.

**Acceptability**,** Appropriateness and Feasibility.** Providers rated the acceptability, appropriateness, and feasibility of TranS-C via three 4-item measures: Acceptability of Intervention Measure (AIM), Intervention Appropriateness Measure (IAM) and Feasibility of Intervention Measure (FIM; Weiner et al., 2017). All measures are rated on a scale from 1 (completely disagree) to 5 (completely agree) and have demonstrated satisfactory known-groups validity, internal reliability, test-retest reliability, and sensitivity to change (Weiner et al., 2017). In the present study, the scales demonstrated excellent internal consistency, as indicated by Cronbach’s alpha (AIM: α = 0.94, IAM: α = 0.95, FIM: α = 0.94).

**Willingness and Confidence.** Providers rated their willingness and confidence to deliver TranS-C. Each construct is rated on a 5-point Likert scale (1 = not willing, 5 = very willing; 1 = not confident, 5 = very confident; Cunningham et al., 2018). For these analyses, these constructs were combined into a single scale (Willingness and Confidence Scale) by taking the average of the two responses.

### Data Analysis

#### Missing Data

Missing data ranged from 0 − 3.2% across measures. A complete case analysis was used as other methods (e.g., multiple imputation) are not indicated when missing data are under 5% (Jakobsen et al., 2017).

#### Substantive Analyses

The first aim was to compare Generation 1 and Generation 2 trainings on the extent of gold standard TranS-C content covered and the extent of gold standard training techniques used by trainers. A one-way multivariate analysis of covariance (MANCOVA) was performed, adjusting for treatment condition. For this aim and the third aim (see below), calculations of gold standard TranS-C content included only shared modules between Standard and Adapted TranS-C (see Table 2). Additionally, the Unhelpful Beliefs about Sleep module was excluded because its content was presented differently between conditions (standalone module in Standard trainings, content points spread throughout in Adapted trainings).

The second aim was to compare Generation 1 and Generation 2 on five provider outcomes (provider knowledge of TranS-C, provider perceptions of the acceptability of TranS-C, provider perceptions of the appropriateness of TranS-C, provider perceptions of the feasibility of TranS-C, and provider willingness and confidence to use TranS-C). A one-way MANCOVA was performed, adjusting for provider theoretical orientation and past training on sleep problems. Theoretical orientation and past training on sleep problems were included as covariates because these constructs may be related to provider attitudes towards and knowledge of EBPTs (Garcia et al., 2020; Nelson & Steele, 2008).

The third aim was to evaluate the effect of the extent of gold standard TranS-C content covered and the extent of gold standard training techniques used on provider outcomes. Two sets of one-way MANCOVAs were performed. The first set examined the effect of the extent of gold standard TranS-C content covered on provider outcomes. The second set examined the effect of the extent of gold standard training techniques used on provider outcomes. Both MANCOVAs were adjusted for generation, treatment condition, provider theoretical orientation and past training on sleep problems.

Partial eta-squared (ηp2) was calculated as a measure of effect size (0.01 = small, 0.06 = medium, 0.14 = large; Cohen, 1988). If the MANCOVA was significant, univariate ANCOVAs were conducted to disentangle the effects. To control Type I error, a Benjamini-Hochberg false discovery rate (FDR; Benjamini & Hochberg, 1995) was applied within each aim, assuming a 5% FDR.

Normality assumptions were checked for both MANCOVAs and ANCOVAs and appropriate precautions were taken when violated. Given normality and linearity violations in at least one variable, all dependent variables were normalized using log transformations. Pillai’s multivariate statistic was used for MANCOVA analyses (Tabachnick & Fidell, 2012) due to the homogeneity of covariances assumption being violated (likely due to imbalanced sample size between generations). Given appropriate precautions were taken to mitigate violations of normality assumptions and MANOVA is fairly robust to deviations from normality (Tabachnick & Fidell, 2012), we proceeded with the planned analyses.

It is also important to note that these data possess a nested structure (e.g., trainings nested within trainers, trainers nested within CMHCs), which violates the MANOVA and ANOVA assumption that all observations are independent. Unfortunately, the sample size of this study precluded the use of multilevel modeling approaches that would be better suited for this data structure (see ‘Conclusions’ for additional discussion of this limitation).

#### Transparency and Openness

This study was pre-registered (osf-registrations-x7v38-v1). All research materials, data, and analysis code are available from the authors upon request. Analyses were conducted using R (v4.3.3; R Core Team, 2021). We report how we determined our sample size, all data exclusions, all manipulations, and all measures in the study.

## Results

Table 3 displays descriptive statistics of the Gold Standard Training Checklist. No deviations from gold standard TranS-C content occurred. Table 4 presents descriptive statistics of observed values for all variables in the analyses below.

**Table 3:** Descriptive statistics of the gold standard training checklist items by generation of Train-the-Trainer

Gold standard training checklist items	Trainings (*N* = 56)	
	Generation 1 (*n* = 32)	Generation 2 (*n* = 24)
	M (SD)	M (SD)
Content Items		
Overview of TranS-C	3.65 (0.88)	3.83 (0.82)
Sleep Diary	3.22 (0.42)	3.29 (0.69)
* Cross-Cutting Modules*		
Case Formulation	2.12 (0.69)	2.29 (1.3)
Behavior Change and Motivation	1.56 (0.62)	1.50 (0.51)
Goal Setting	3.34 (1.15)	3.17 (0.96)
* Core Modules*		
Establishing Regular Sleep-Wake Times	4.06 (0.62)	4.38 (0.65)
Learning a Wind-down Routine	4.66 (0.48)	4.88 (0.34)
Learning a Wake-up Routine	5.0 (0.0)	5.0 (0.0)
Improving Daytime Functioning	4.84 (0.45)	4.83 (0.48)
Maintenance of Behavior Change	5.0 (0.0)	4.79 (1.02)
* Optional Modules*		
Reducing Sleep-related Worry and Vigilance	4.56 (0.5)	4.79 (0.41)
Technique Items		
Agenda Setting	1.78 (0.83)	2.38 (0.65)
Behavioral Rehearsal (Role-Play) * # of role plays*:	0.03 (0.18) *0.03 (0.18)*	0.04 (0.2) *0.04 (0.2)*
Trainer Modeling * # of trainer modeling instances*:	2.13 (0.55) *5.22 (1.1)*	1.75 (0.61) *3.83 (1.2)*
Socratic Questioning	1.25 (0.51)	1.17 (0.48)
Positive Reinforcement/Praise	2.88 (0.34)	2.21 (0.66)
Discussion to Promote Self-Reflection	0.56 (0.91)	1.63 (0.82)
Critical Collaborative Inquiry * # of breakout groups*:	0.19 (0.4) *0.19 (0.4)*	0.17 (0.48) *0.08 (0.28)*
Other Activities to Promote Active Learning	2.13 (0.55)	2.67 (0.48)
Trainee Response and Engagement	2.19 (0.74)	2.79 (0.41)
Deviations from Gold Standard TranS-C	0.0 (0.0)	0.0 (0.0)

Non-transformed variables are displayed. *Italicized* variables refer to raw count variables. All remaining variables reflect extensiveness ratings, ranging from 0–5 for TranS-C Content Items and 0–3 for Technique Items. Only shared content between Standard and Adapted TranS-C are presented here (see ‘Data Analysis’ for more details)

**Table 4:** Descriptive statistics of all observed variables

Variable	Generation 1 M (SD)	Generation 2 M (SD)
*Gold Standard Training Checklist*		
TranS-C Content	3.82 (0.24)	3.89 (0.25)
Training Techniques	1.46 (0.31)	1.64 (0.25)
*Provider Outcomes*		
Knowledge	5.53 (2.00)	5.02 (2.44)
Acceptability	4.68 (0.44)	4.77 (0.38)
Appropriateness	4.67 (0.47)	4.82 (0.33)
Feasibility	4.59 (0.47)	4.64 (0.53)
Willingness and Confidence	4.38 (0.51)	4.35 (0.55)

Outliers were present in provider outcomes (see Fig. 2). One outlier was deemed a data entry error and excluded. All others were retained for analyses (Leys et al., 2019). Given the susceptibility of MANCOVA to outliers (Tabachnick & Fidell, 2012), sensitivity analyses were performed excluding extreme outliers. Extreme outliers were identified using the ‘rstatix’ package in R (Kassambara, 2023). Significant changes to the original findings were minimal and are discussed below.

### Effect of Generation on Gold Standard Training Elements (Aim 1)

**Fig. 1:**
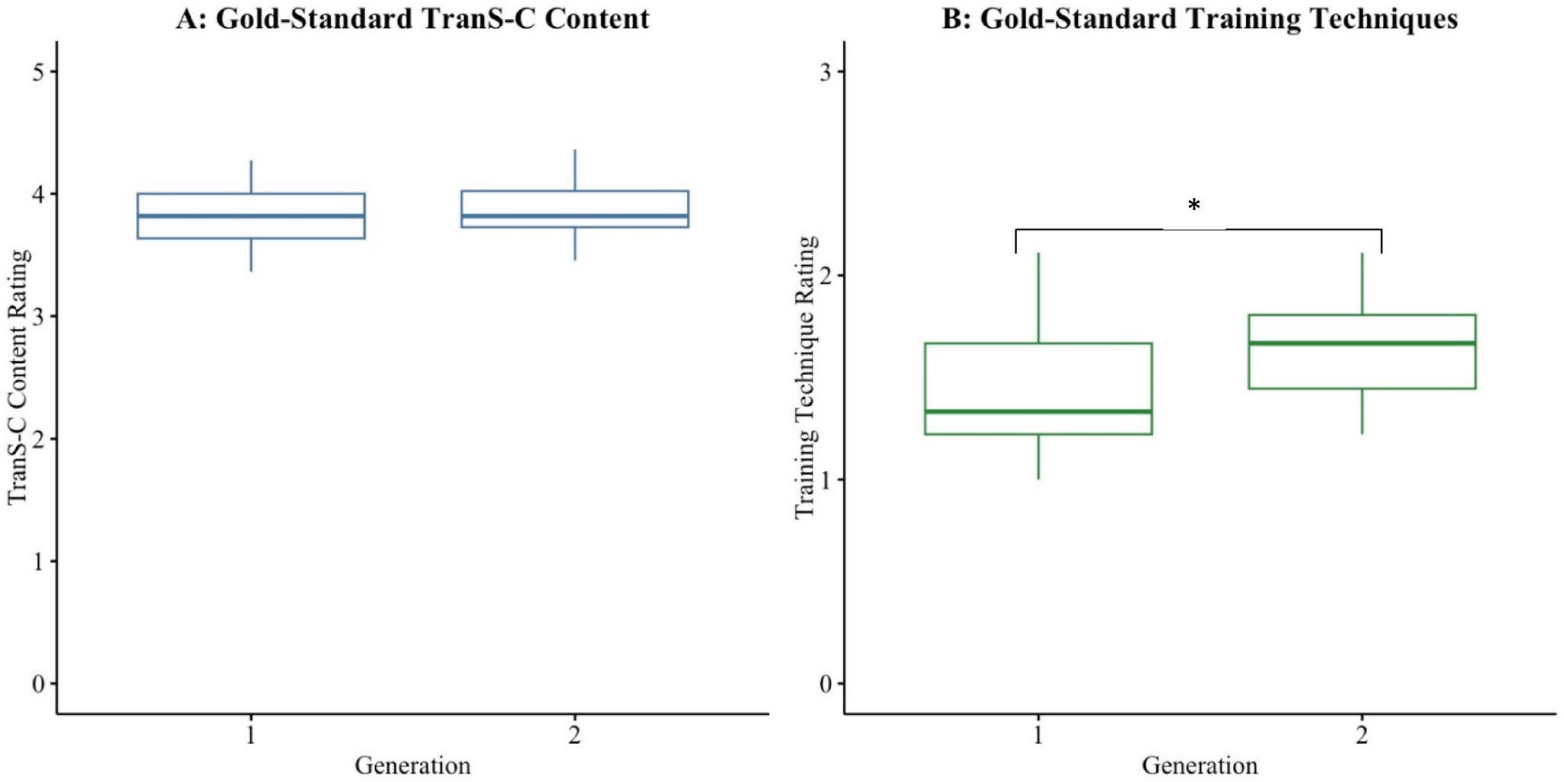
Aim 1 - Multi-panel Boxplots. Possible scores for TranS-C Content overall extensiveness ratings ranged from 0–5 (Graph A). Possible scores for Training Technique overall extensiveness ratings ranged from 0–3 (Graph B). No outliers were detected. *Denotes significance *p* < 0.05 after Benjamini-Hochberg false discovery rate correction

Results are displayed in Table 5; Fig. 1. A MANCOVA was used to examine the effect of generation on the extent of gold standard TranS-C content covered and the extent of gold standard training techniques used, adjusting for treatment condition. The main effect was significant (F(2, 52) = 5.0, Pillai’s trace = 0.15, *p* = 0.015), suggesting the linear combination of the training content and technique variables was significantly different between Generation 1 and Generation 2 trainings. The effect size was large (*η*^2^ partial *=* 0.15). Follow-up univariate ANCOVA tests, adjusting for treatment condition, indicated that there was no significant effect of generation on content covered (F(1,53) = 2.24, *η*^2^ partial *=* 0.04, *p* = 0.14). However, there was a significant effect of generation on training techniques used (F(1,53) = 6.80, *η*^2^ partial *=* 0.11, *p* = 0.012), such that local trainers in Generation 2 delivered gold standard training techniques to a greater extent compared to expert trainers in Generation 1. These findings remained significant after the Benjamini-Hochberg FDR correction.

**Table 5:** Results of MANCOVA examining the effect of generation (1 vs. 2) on gold standard training content and techniques (Aim 1)

Variable	F	*p*	Partial Eta Squared
*Gold Standard Training Checklist*			
TranS-C Content	2.24	0.14	0.04
Training Techniques	6.80	0.012*	0.11
Pillai’s Trace = 0.15 [F(2, 52) = 5.00; *p* = 0.015*]			

MANCOVA = multivariate analyses of covariance

*Significant after within-domain Benjamini-Hochberg false discovery rate correction

### Effect of Generation on Provider Outcomes (Aim 2)

**Fig. 2:**
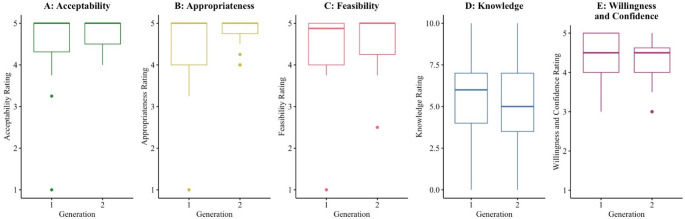
Aim 2 - Multi-panel Boxplots. Outliers were carefully inspected for data entry errors. One outlier was identified as erroneous and was excluded from analyses. Possible scores for the Acceptability, Appropriateness, and Feasibility of TranS-C (Graphs A-C) and Willingness and Confidence to use TranS-C (Graph E) ranged from 1–5. Possible scores for Knowledge of TranS-C (Graph D) ranged from 0–10

Figure 2 displays variables using boxplot graphs. MANCOVA was used to examine the effect of generation on provider outcomes, adjusting for provider theoretical orientation and past training on sleep problems. The main effect for generation was not significant (F(5, 141) = 2.00, *η*^2^ partial *=* 0.07, Pillai’s trace = 0.07, *p* = 0.071), suggesting the linear combination of provider outcomes was not significantly different between Generation 1 and Generation 2.

Results of sensitivity analyses (excluding extreme outliers) are presented in Table 6. The main effect (MANCOVA) for generation was significant (F(5, 137) = 2.00, *η*^2^ partial *=* 0.08, Pillai’s trace = 0.08, *p* = 0.04). Follow-up univariate ANCOVAs revealed a significant effect of generation on provider ratings of acceptability (F(1, 150) = 5.10, *η*^2^ partial *=* 0.03, *p* = 0.03), appropriateness (F(1, 150) = 10.89, *η*^2^ partial *=* 0.07, *p* = 0.001), and feasibility (F(1, 150) = 3.93, *η*^2^ partial *=* 0.03, *p* = 0.049) of TranS-C. In other words, providers trained by local trainers in Generation 2 rated TranS-C to be more acceptable, appropriate, and feasible compared to providers trained by expert trainers in Generation 1. However, only one of these findings (provider ratings of appropriateness of TranS-C) remained significant after the Benjamini-Hochberg FDR correction.

**Table 6:** Results of MANCOVA examining the effect of generation (1 vs. 2) on provider outcomes (Aim 2)– Extreme outliers removed

Variable	Generation 1 M (SD)	Generation 2 M (SD)	F	*p*	Partial Eta Squared
*Provider Outcomes*					
Knowledge	5.53 (2.00)	5.27 (2.27)	0.23	0.63	0.00
Acceptability	4.68 (0.44)	4.84 (0.32)	5.10	0.03	0.03
Appropriateness	4.67 (0.47)	4.91 (0.20)	10.89	0.00*	0.07
Feasibility	4.59 (0.47)	4.75 (0.38)	3.93	0.049	0.03
Willingness and Confidence	4.38 (0.51)	4.37 (0.52)	0.01	0.91	0.00
Pillai’s Trace = 0.08 [F(5, 137) = 2.00; *p* = 0.04]					

MANCOVA = multivariate analyses of covariance

*Significant after within-domain Benjamini-Hochberg false discovery rate correction

### Effect of Gold Standard Training Elements on Provider Outcomes (Aim 3)

The first MANCOVA examined the effect of the extent of gold standard TranS-C content covered on provider outcomes, adjusting for generation of TTT, treatment condition, provider theoretical orientation, and past training on sleep problems. The main effect for gold standard TranS-C content was not significant (F(5, 79) = 0.48, *η*^2^ partial *=* 0.03, Pillai’s Trace = 0.03; *p* = 0.79), suggesting the linear combination of provider outcomes was not significantly different based on the extent of gold standard TranS-C content delivered by trainers.

The second MANCOVA examined the effect of the extent of gold standard training techniques used on provider outcomes, adjusting for the same variables. The main effect for gold standard TranS-C techniques was not significant (F(5, 79) = 0.40, *η*^2^ partial *=* 0.02, Pillai’s Trace = 0.02, *p* = 0.87), suggesting the linear combination of provider outcomes was not significantly different based on extent of gold standard TranS-C techniques delivered by trainers.

No significant changes to these findings were found in sensitivity analyses.

## Conclusions

The overarching goal of this study was to progress knowledge of TTT, a popular yet understudied training structure theorized to produce sustainable EBPT training programs. Specifically, this study sought to evaluate whether training quality and related provider outcomes were maintained across two generations of TTT. In this study, two variations of TranS-C (Standard and Adapted) were implemented via TTT in CMHCs for adults with SMI and sleep and circadian problems.

The first aim was to compare Generation 1 and Generation 2 trainings on the extent of gold standard training elements (TranS-C content and training techniques) delivered by trainers. We hypothesized that Generation 1 trainings would outperform Generation 2 trainings due to a poor transfer of knowledge from expert trainers to local trainers. Contrary to our hypothesis, local trainers delivered more gold standard training techniques compared to expert trainers, while maintaining the same delivery of gold standard training content. Put another way, local trainers used more active learning strategies than expert trainers without sacrificing the amount of content delivered. These findings are quite promising, as they not only a signal successful transfer of knowledge from expert trainers to local trainers, but they also raise the possibility of training quality *improving* in subsequent generations of TTT led by local trainers.

We offer several possible explanations for these findings. First, the relationship between trainers and trainees may have impacted the ease with which trainers delivered training techniques. Generation 2 local trainers had pre-established relationships with trainees, whereas Generation 1 expert trainers were “outsiders.” Thus, local trainers may have been more effective in initiating and elongating active learning opportunities that encouraged trainee engagement and participation. Indeed, upon inspecting the means of each training technique used between local and expert trainers (see Table 3), many of the techniques used more extensively by local trainers invite or require participation from trainees (e.g., discussion to promote self-reflection, trainee response and engagement).

Second, the local trainers may have harnessed their understanding of and first-hand experiences within the CMHC context to enrich active learning discussions. This is aligned with the Dynamic Sustainability Framework (Chambers et al., 2013), which rejects the idea of “program drift” and instead posits that the maximal benefit of an intervention (in this case, the EBPT training) can only be realized in ongoing development within the implementation context. In other words, perhaps implementation of trainings in community settings works best when they are continuously refined and improved as they are sustained.

Third, it is possible that local trainers possessed certain characteristics (e.g., charisma, warmth; Boyd et al., 2017) or pre-existing skills (e.g., active listening, emotion regulation) that made them especially effective at facilitating active learning opportunities (Ng & Lam, 2015). Indeed, there have been calls in the literature for increased attention to the impact of trainer characteristics on EBPT implementation outcomes, particularly when training is delivered virtually (Larson et al., 2022).

Finally, self-selection bias must be considered. During Generation 1, providers were strongly encouraged or even mandated by CMHC leadership to attend trainings, whereas providers typically volunteered to attend Generation 2 trainings. This may have resulted in a more motivated composition of Generation 2 trainees, facilitating trainer use of training techniques.

It is worth noting that some training techniques were rarely used across both generations, indicating they were difficult to incorporate into trainings (see Table 3 for descriptive statistics). Namely, behavioral rehearsal (role-play) and critical collaborative inquiry (breakout groups) were the least used by both expert and local trainers. Despite the strong literature behind their effectiveness (e.g., Cross et al., 2007), role-plays are often underutilized which may be due to the performance anxiety they can evoke (Beidas et al., 2014). Infrequent breakout groups may reflect time constraints given these were typically slated to occur at the end of trainings in the current study. The lack of role-plays in trainings is particularly concerning, as the literature consistently reports a link with improved EBPT adoption, skill, and fidelity (e.g., Beidas & Kendall, 2010; Frank et al., 2020; Triplett et al., 2020).

The second aim was to compare Generation 1 and Generation 2 on a post-training assessment that evaluated provider knowledge of TranS-C, provider perceptions of the acceptability, appropriateness, and feasibility of TranS-C, and provider willingness and confidence to use TranS-C (“provider outcomes”). Contrary to our hypothesis, there were no significant differences between generations on provider outcomes. In sensitivity analyses, Generation 2 providers outperformed Generation 1 providers on three outcomes (acceptability, appropriateness, and feasibility of TranS-C), though only one remained significant after correction. Though this is a weak signal, it suggests that local trainers may foster more positive provider perceptions of EBPTs than expert trainers, which in turn might predict stronger EBPT adoption (Proctor et al., 2023). Again, these findings are highly encouraging for TTT.

The hypothesized advantages for local trainers discussed above may be relevant here. For instance, local trainers may have used their experiences in CMHCs to make subtle adaptations to trainings to increase the significance and practicality of TranS-C (e.g., perhaps they gave more relevant examples). Pre-existing relationships with local trainers may have influenced trainees to rate TranS-C more favorably. As providers tended to volunteer for Generation 2 trainings, they may have viewed EBPTs or sleep treatment more positively prior to training. It is also possible that providers found local trainers to have more credibility, an important trainer characteristic perceived by trainees (Boyd et al., 2017), with respect to their endorsement of TranS-C in the CMHC setting. While prior research has reported that trainees tend to rate expert trainers as having more credibility than local trainers with respect to their general intelligence and qualifications (e.g., Triplett et al., 2020), perhaps local knowledge of the implementation setting is more salient when considering EBPT acceptability, appropriateness, and feasibility. It is imperative that future research articulate the advantages of local trainers, such that they can be harnessed to optimize provider outcomes in TTT.

The third aim was to evaluate the effect of the extent of gold standard TranS-C content covered and the extent of gold standard training techniques used on provider outcomes. Contrary to our hypotheses, there was no significant effect of these training elements on provider outcomes. One possibility is that the provider outcomes measured here are not significantly impacted by an increased delivery of gold standard content and training techniques. For instance, the body of literature linking active learning strategies to optimal provider outcomes has historically examined provider adherence and skill post-training (Beidas & Kendall, 2010), which are conceptually distinct from the outcomes of the present study. Another possible explanation is that the restricted variability on both gold standard content [range(0–5): 3.36–4.36, mean(sd): 3.85(0.25)] and technique total scores [(range(0–3): 1-2.11, mean(sd): 1.54(0.3)] limited our ability to detect an effect in this sample.

Relatedly, while quality delivery of both EBPT content and active learning techniques are considered vital, the ideal dosage of these elements is ill-defined. As training time is often limited in routine practice settings (Swain et al., 2010), it is important for future research to articulate the optimal frequency, intensity, and duration of these elements across the training period. In this vein, it may be fruitful to explore the use of standardized, pre-recorded trainings to better manipulate and compare the variation in elements used. Indeed, recent systematic reviews (Mikkonen et al., 2024; Valenstein-Mah et al., 2020) have highlighted that asynchronous or self-paced learning (i.e. self-directed and individually paced by each trainee) evidence non-inferior outcomes compared to conventional, ‘live’ training methods and hold potential to increase access and scalability. These methods may be worth exploring to (a) cleanly examine the optimal ratio of content to active learning activities and (b) ensure the inclusion of powerful strategies such as role plays that are often neglected in live trainings.

These findings must be considered in the context of the limitations of this study. First, the Gold Standard Training Checklist and the Knowledge Test are novel measures created for this study and thus the results must be interpreted with caution. Promisingly, interrater reliability of both measures was excellent, with ICCs ranging from 0.875 to 0.99. Strengths of the Gold Standard Training Checklist include the rigor of its development, including a comprehensive review of the literature and TranS-C manual to define gold standard elements, and the consensus scoring approach of blinded coders maximized reliability of the final scores. The low variation in scores across training videos (see Table 3) may signal construct validity, given all trainers used the same slide deck, with small improvements instituted overtime. That said, future research is needed to continue establishing validity and reliability of this checklist. Regarding the Knowledge Test, face validity was confirmed by several experienced TranS-C providers, following other studies (e.g., Kuo et al., 2021). However, the measure would be strengthened by future research demonstrating content validity (e.g., by giving the test to trained vs. untrained providers) and sensitivity to change (e.g., by administering the test pre- and post-training).

Second, the Gold Standard Training Checklist captures both thoroughness and frequency of content and technique delivery in a single “extensiveness” rating, following prior research (Hogue et al., 1996). While this method was selected to maximize reliability and feasibility of coding, it may have reduced our ability to detect differences between trainings. Indeed, as mentioned above, variability was restricted on both content and technique total scores. While this may signal construct validity, it is also possible this scoring method was not sufficient to detect the smallest unit of meaningful change.

Third, the impact of the length of each training was not examined. However, training length was essentially captured by adjusting for treatment condition, as each condition had a standardized training length (six hours for Standard TranS-C vs. four hours for Adapted TranS-C). Additionally, coders were instructed to take the length of the training into account (e.g., “use judgment based on the length of training;” see Online Resource 1).

Fourth, although these data have a nested structure (i.e. trainings within trainers, trainers within CMHCs), this study did not utilize multilevel modeling. Due to several trainings being led by multiple trainers for trainees across multiple CMHCs, cross-classified, multiple membership multilevel modeling would be needed to fully account for this variation. These complex data structures require sample sizes not achieved by this study (Chung et al., 2018). Thus, the findings reported in this study do not account for variation across trainers and CMHCs. Relatedly, it would be valuable to examine the influence of trainer demographic characteristics (e.g., caseload, degree, years of experience) on training quality in future research. Trainer characteristics were not included as covariates in the present analyses due to many trainings being led by multiple trainers.

These limitations notwithstanding, the results of the present study suggest a successful transfer of knowledge from Generation 1 expert trainers to Generation 2 local trainers. This begs the question: How was this transfer facilitated? Leberman and colleagues (2006) once said that “transfer of learning is best accomplished when it meets the specific needs of the context.” Put another way, education and training is most effective when prioritizing practical, “on the job” application (Haskell, 2001). It is probable that the present study succeeded in both meeting the needs of the CMHC context and providing enough scaffolding for the nuts-and-bolts application. Indeed, the TranS-C trainings were developed specifically for the CMHC context, considering the needs of its providers and population of patients served. Furthermore, both Generation 1 and Generation 2 trainings were conducted in the same CMHCs and were delivered largely via the same medium^1^ (Zoom). Training of local trainers was also conducted over Zoom, using the same (or very similar) slide deck with which they were originally trained. The slides included detailed presenter notes and reminders to initiate training techniques. These “similar elements” may have been enough to facilitate successful transfer. Active learning strategies used by the UC Berkeley team when training the local trainers may have been helpful as well (e.g., training in public speaking, behavioral rehearsal, positive reinforcement, individualized feedback). Future research is needed to disentangle these components and identify the active ingredients of successful transfer between generations of trainers.

This raises another question: Will the transfer of knowledge of the EBPT generalize beyond the training? As discussed earlier, gold standard EBPT trainings initiatives are comprised of multiple components, including ongoing consultation and supervision alongside the training itself (e.g., Valenstein-Mah et al., 2020, Barnett et al., 2021). Future research ought to test whether the quality of consultation and supervision is (a) established in Generation 1 and (b) maintained, or ideally, improved upon in Generation 2 to support sustained, high-quality delivery of the EBPT in a TTT structure (e.g., Barnett et al., 2021). Because consultation and supervision require trainers to operate almost exclusively “off script,” it may offer an ideal setting in which to test local trainers’ generalized knowledge of EBPTs across generations of TTT.

Taken together, this study supports the potential of the TTT approach to sustain training quality and select provider outcomes to a second generation of local trainers. Moreover, the findings highlight the potential for Generation 2 local trainers to outperform Generation 1 expert trainers. This speaks to the possible benefits of placing trainings in the hands of individuals with context-specific knowledge and pre-existing relationships with trainees (Chambers et al., 2013; Yarber et al., 2015). Considering these promising findings, future research ought to (a) identify the optimal dosage of gold standard training elements relative to training length, (b) investigate the factors driving variation among standardized trainings (e.g., time limitations, trainer characteristics), (c) confirm transfer of knowledge to other training activities beyond the training workshop, and (d) extend investigations of training quality to a third generation of trainers (and beyond).

## Electronic Supplementary Material

Below is the link to the electronic supplementary material.

Supplementary Material 1

## Data Availability

All research materials, data, and analysis code are available from the authors upon request.

## Footnotes

1 The first two Generation 1 trainings were held in person before the onset of the COVID-19 pandemic. All subsequent Generation 1 trainings, and all Generation 2 trainings, were held via Zoom.
