# Supplementary material for: An Examination of Training Quality and Provider Outcomes Across Two Generations of Train-the-Trainer
Source: Adm Policy Ment Health. 2025 Aug 21;52(5):966–82. doi: 10.1007/s10488-025-01463-w (PMC12449322; doi:10.1007/s10488-025-01463-w)
Supplement: Supplementary file 1 — Supplementary Material 1 [file 10488_2025_1463_MOESM1_ESM.pdf]

SUPPLEMENTAL FILE

An Examination of Train-the-Trainer across Two Generations: A "Scale-up Penalty" on Training and Provider Outcomes?

Catherine A. Callaway, MA, Joshua M. Varghese, Emma R. Agnew, LCSW, Laurel D. Sarfan, PhD, Allison G. Harvey, PhD

Corresponding Author: Allison G. Harvey, University of California, Berkeley, aharvey@berkeley.edu

Gold Standard Training Checklist

Coder Instructions

1. On *Content* Extensiveness Ratings:
    a. Extensiveness ratings are based on the total number of bullet points covered. Subject headings with no bullet point underneath DO count as bullet points. Subject headings with bullet points underneath DO NOT count as bullet points themselves.
    b. While watching the training videos, highlight the bullet points that are covered by the trainer. After you are done, calculate the percentage of bullet points that are covered in each module, and convert the percentage to extensiveness ratings **(0% -> 0, 1%-20% -> 1, 21%-40% -> 2, 41%-60% -> 3, 61%-80% -> 4, and 81%-100% -> 5).**
    c. It is common that a bullet point is *partially covered* and you may wonder if you should code it as covered or not. This is a gray area – go with your gut! This is why we have multiple coders who will come to a consensus in meetings. You will develop a better "nose" for this as you go.
2. On *Content* Points that are Modeled:
    a. If a trainer *models* a treatment point, this COUNTS as covering the treatment point.
        i. Example #1: One content point for the Behavior Change and Motivation module is "Supplement agenda with humorous/enjoyable add-ons (videos, articles)." The trainer may show the clinicians enjoyable videos (e.g., video about snoozing) throughout the training, but they may not explicitly state that the clinicians should show these videos/articles to their patients or relate showing these videos to the concept of motivation. This should still be counted as teaching that bullet point.

    ii. Example #2: If the trainer goes through a Pro/Con worksheet, this should count as teaching/modeling the *Pros and cons of making a change vs. not making a change* in the Behavior Change and Motivation Cross-Cutting Module.
  b. Many treatment points under the Sleep Diary module and cross-cutting modules are modeled this way; be careful not to miss them. Pay particular attention to the trainer interacting with providers: when asking about their sleep or discussing a case example, the trainer may model cross-cutting skills.

3. On Deviations from Gold Standard TranS-C Material:
    a. This is not likely to happen often, but it is important to catch if and when it does. If the trainer is reading directly from a page in the workbook, you can be sure it is TranS-C material. If the trainer begins discussing material that gives you pause (e.g., “lavender spray helps with your sleep”), note it here and we will discuss it together in the consensus meeting.
4. On Recording Mishaps:
    a. Sometimes, it becomes clear that the recording started late or was stopped early (e.g., the trainer is startled when it turns on and they are in the middle of teaching a content area). Please note here any instances you suspect this happened.
5. On *Teaching* Techniques:
    a. See the technique table for coding tips to help you spot each teaching technique when it occurs, and for suggested anchors for extensiveness ratings (ranging from 0-3).
        i. Example: For Socratic Questioning, 0 corresponds to “Socratic questioning never used,” and 3 corresponds to “Socratic questioning used frequently throughout training.” There is no set number, as the trainings vary in length. After you have watched multiple trainings, and with the guidance of *(name of consensus meeting leader)* you will get a sense of what “frequently” and “never used” refer to, and correspondingly, what a 1 or 2 rating should refer to.
    b. Record frequency counts for Behavioral Rehearsal, Training Modeling, and # of breakout groups (these are highlighted under Critical Collaborative Inquiry) to aid in consensus discussions. If you have questions about what counts for each of these categories, jot it down for discussion as well.
    c. Please use the teaching techniques column in the first table (content table) to record preliminary 0-3 ratings and frequency counts for each teaching technique as you go through *each* content section. This is merely to help you keep track of each technique as you watch these lengthy trainings. Use the preliminary ratings to inform your final extensiveness ratings (record final ratings in the technique table) and frequency counts.

Table 1: Gold Standard TranS-C Content

Definition of a key treatment point: *the key content and skills taught during training that the trainer thinks are important for the sleep coach to understand in order to provide effective sleep coaching to patients.*

	CONTENT ITEMS	EXTENSIVENESS RATINGS (0-5)
OVERVIEW OF TRANS-C<br>(16 treatment points)	Overview of TranS-C<br>● *Flexibility (education on 4 core, 7 optional, 4 cross-cutting)*<br>● *Order of modules: Start with routines (Module 1B-C), then can mix up, end with Maintaining Gains*<br>● *Length (50-minute sessions, 4-10 are typically sufficient)*<br>● *Patient population (variety of serious mental illness and sleep disorders)*<br>● *Providers who can deliver TranS-C (no degree needed, CBT background preferred)*<br><br>Research behind TranS-C / Empirical Evidence<br>● *Benefits of healthy sleep*<br>● *Consequences of unhealthy sleep*<br>● *Relationship between serious mental illness and sleep*<br><br>Session Structure<br>● *Set an agenda*<br>● *Review previous session content*<br>● *Review and comment on sleep diary*<br>● *Review goals/homework from last session*<br>● *Go over next/most relevant module*<br>● *Set goals/homework for next week*<br>● *Ask patient to summarize session*<br>● *Ask for feedback on session*	
SLEEP DIARY	How to introduce/teach sleep diary to patient	

(17 treatment points)	• *Go through last night of sleep to train patient on how to fill out the sleep diary*<br>• *Rationale: how we plan treatment and track progress*<br>• *Use felt sense – no clock watching*<br>• *Filling out the sleep diary acts as its own intervention*<br>• *Adaptable to each person's needs*<br>• *Ideally start before sleep treatment begins*<br><br>How to handle non-compliance (not turning in sleep diaries)<br>• *For therapist: it is normal!*<br>• *Do last night of sleep, ask if it was typical*<br><br>How to calculate variables and interpret as therapist<br>• *TIB (Time in Bed) calculation*<br>• *TST (Total Sleep Time) calculation (7-8 hours recommended for adults)*<br>• *SE (Sleep efficiency) calculation (85%, 80% for older adults)*<br>• *MST (midpoint of sleep) calculation (MST should be between 2-4AM ideally)*<br><br>What to look for when reviewing sleep dairy<br>• *Irregularity*<br>• *Long SOL (Sleep Onset Latency)*<br>• *Long WASO (Wakefulness After Initial Sleep Onset)*<br>• *Snoozing*<br>• *Naps – check for frequency and duration*	
CASE FORMULATION (14 treatment points)	*(see sleep diary in sleep assessment)*<br><br>Sleep "Snapshot"<br>• *Specific, typical, recent night when a problem was evident*<br>• *Focus on modifiable factors that maintain sleep problems*<br><br>Introduce CBT model<br>• *Thoughts related to sleep*	

	● *Feelings related to sleep*<br>● *Behaviors related to sleep*<br>● *Thoughts, feelings, and behaviors all influence each other*<br><br>Assess which optional modules might be needed<br>● *1 – if sleep efficiency is less than 85% on average across the week (or 80% for older adults)*<br>● *2 – if patient is getting too much sleep or spending too much time in bed (more than 9-10 hours on average)*<br>● *3 – if bedtime is earlier/later than preferred by patient, or later than 2am, or midsleep times outside of 2-4am range*<br>● *4 – if patient shares sleep related worry or rumination – assessment on pg. 122*<br>● *5 – if patient is having difficulty adapting to use of CPAP*<br>● *6 – if environmental factors are interfering with sleep*<br>● *7 – if patient experiences frequent nightmares that cause distress*<br><br>If unsure, collect data!	
BEHAVIOR CHANGE & MOTIVATION (15 treatment points)	Behavior Change<br>● *Normalize that change is difficult and takes time (8+ tweaks needed)*<br>● *Validate if patient is struggling to enact changes (many sleep-interfering behaviors are rewarding)*<br>● *Implementation intentions (how, when, and where will the new behavior be implemented e.g., "How can YOU fit this in your routine")*<br>● *Discuss real and perceived barriers*<br>● *Repetition and practice are essential*<br>● *Promote patient remembering the plan ("What would help you remember to do this?")*<br>● *Conduct functional/chain analysis if change is not being made (thoughts, feelings, behaviors)*<br><br>Motivation<br>● *Find intrinsic motivators specific to patient*	

	● *Discuss how sleep material is relevant to patient's life/goals*<br>● *Motivational interviewing approach (Non-judgmental, empathy, collaboration)*<br>● *Ask open-ended questions to elicit patient's reasons for change ("change talk")*<br>● *Pros and cons of making a change vs. not making a change*<br>● *Mental contrasting: desired future with present reality*<br>● *Recognize/reinforce ANY small amount of progress*<br>● *Supplement agenda with humorous/enjoyable add-ons (videos, articles)*	
GOAL SETTING<br>(6 treatment points)	Goal setting<br>● *Set goals for night AND day*<br>● *Goals should be realistic, specific, measurable*<br>● *Look out for unhelpful/unrealistic expectations (e.g., "deep sleep" or "uninterrupted sleep") – opportunity for education*<br>● *Set goals in session 1 or 2, but readjust as you go (utilize patient feedback and sleep diary)*<br>● *Initial goals are long range; set small steps towards long range goals week to week*<br>● *Add rows to sleep diary to monitor/track goals*	
Core Modules		
1A- ESTABLISHING REGULAR SLEEP-WAKE TIMES<br>(27 treatment points)	Rhythmic bodies<br>● *We have rhythmic bodies*<br>● *Every cell and organ in the body has its own clock*<br>● *We move too fast & often medicate these rhythms*<br><br>Regular sleep/wake times<br>● *Regular sleep/wake times are important for rhythms*<br>● *Irregular sleep/wake times interrupts circadian rhythm (puts body in jet-lagged state)*<br>● *Brain is like temporal orchestra*<br>● *1 hour variation in bed-wake times is what to aim for*	

	Sleep stages<br>● *The different sleep stages are important for different basic life functions*<br>● *REM is important for emotional processing*<br>● *NREM is important for growth and repair*<br>● *More deep at the beginning, more light at the end*<br><br>Typical/normal night of sleep<br>● *Brief awakenings (1-3)*<br>● *We go back into the stage we need most*<br>● *Deep and light stages*<br>● *20-30 min to fall asleep/go back to sleep*<br>● *Grogginess up to an hour after waking*<br>● *TST (total sleep time) needed varies as we age*<br><br>Two process model of sleep<br>● *Homeostatic pressure to sleep increases every moment we spend awake and is discharged when we sleep*<br>● *Naps discharge our homeostatic pressure*<br>● *Circadian rhythm is roughly 24 hours and needs to be synchronized by external cues*<br>● *Circadian rhythm helps us be awake during the day and sleepy at night*<br><br>Melatonin / SCN (Suprachiasmatic Nucleus) / Light & dark cues<br>● *SCN is our master clock (orchestra conductor) and keeps rhythms in sync*<br>● *SCN controls melatonin production*<br>● *Melatonin is a hormone naturally produced by our brain and makes us feel sleepy*<br>● *Melatonin can only be released in the dark (light suppresses melatonin release)*<br><br>Set realistic bed and wake times the patient can stick to all 7 days of the week<br>Regularize other daily rhythms as well (meals, exercise, socializing)	

1B- LEARNING A WIND-DOWN ROUTINE<br>(4 treatment points)	Sleeping and waking are like dimmer switches<br><br>Relaxing activities in the hour before bed helps us wind down for sleep<br>Dim light<br>● *Dim light or darkness for the hour before bed releases melatonin and promotes sleepiness*<br>● *Limit light from screens*	
1C- LEARNING A WAKE-UP ROUTINE<br>(11 treatment points)	Morning light is very important<br><br>Regular wake-up time serves as an anchor for body rhythms<br><br>Morning grogginess (sleep inertia) is normal!<br><br>RISE UP<br>● *RISE UP strategies make us feel more awake and alert in the morning*<br>● *Refrain from snoozing*<br>● *Increase activity*<br>● *Shower/splash with cool water*<br>● *Exposing to light (sunlight)*<br>● *Upbeat music*<br>● *Phone a friend / Social activity*<br>● *Don't need to do each one as the patient! Important to pick a few and do consistently*	
2- IMPROVING DAYTIME FUNCTIONING<br>(11 treatment points)	Circadian rhythm<br>● *Circadian rhythm fluctuates throughout the day and so does our energy*<br>● *Post lunch dip is normal*<br><br>Staying active	

	• *Staying active during the day helps us feel more energized than restful behaviors*<br>• *Energy levels are elastic (the more we expend, the more we get)*<br>• *Energy generating experiment*<br><br>Napping<br>• *It is best to eliminate naps, or reduce/move early in the day*<br>• *Before 3:00pm for 20 minutes or less*<br><br>Sleep-interfering drinks<br>• *Drinks containing alcohol and caffeine can interfere with sleep*<br>• *Stop having caffeine after noon – sticks around for a LONG time, long half life*<br>• *Limit alcohol intake*<br><br>Use the bed for sleep/sex only to build a strong association between the bed and sleep	
CORRECTING UNHELPFUL SLEEP-RELATED BELIEFS<br>(8 treatment points)	Beliefs and attitudes about sleep<br>• *Our beliefs and attitudes about sleep can contribute to sleep problems*<br>• *Keep realistic expectations for sleep*<br>• *Don't always blame sleeplessness*<br>• *Rethink the causes of sleep problems*<br>• *Avoid "trying" to sleep*<br>• *Avoid panicking after poor sleep*<br>• *Avoid emphasizing sleep*<br>• *Develop tolerance to sleep loss*	
MAINTENANCE OF BEHAVIOR CHANGE<br>(2 treatment points)	Practicing what you've learned in sleep coaching in your daily life going forward requires planning and thoughtfulness<br><br>Encountering setbacks is normal and we can reduce their impact by planning for them	

Optional Modules		
1- IMPROVING SLEEP EFFICIENCY<br>(7 treatment points)	Vicious cycle of worrying and wakefulness<br><br>Stimulus control<br>● *Go to bed when feeling sleepy*<br>● *Get out of bed if unable to sleep in 15-20 minutes, return to bed when sleepy (repeat as needed)*<br>● *Avoid checking the clock*<br><br>Sleep restriction<br>● *Restrict the amount of time you spend in bed to the average amount you are sleeping*<br>● *Build an association between the bed and sleep*<br>● *How to set a sleep window (pg. 149)*	
2- REDUCING TIME IN BED<br>(4 treatment points)	Consequences of too much sleep<br>● *Feel worse on waking (worsens groggy feeling)*<br>● *Have fragmented sleep*<br>● *Higher risk of cardiovascular disease, depression*<br>● *Experience disruptions in social life, daily activities (like work or school) and overall motivation*	
3- DEALING WITH DELAYED OR ADVANCED PHASE<br>(11 treatment points)	Bedtime/Waketime<br>● *Range for a healthy wake-time is 6-8:30am; healthy bedtime is 9:30pm-12am*<br>● *Delayed phase: goes to bed later and wakes up later*<br>● *Advanced phase: goes to bed earlier and wakes up earlier*<br><br>Adjust sleep cycle in desired direction by 20-30 minutes each week<br><br>Special instructions for delayed phase…<br>● *Emphasize regular wake up time*<br>● *Activity is crucial during the morning and mid-day*	

	• *Avoid caffeine after midday*<br>• *Get sunlight/bright light in the morning around the same time*<br><br>Special instructions for advanced phase…<br>• *Emphasize light exposure into the evening to delay melatonin production*<br>• *Craft evening activities/social connection after dinner and before wind down*<br>• *Can try small amount of caffeine later in the day*	
4- REDUCING SLEEP-RELATED WORRY AND VIGILANCE<br>(12 treatment points)	Worries<br>• *Normalize everyone's brains come back to sticky worries*<br>• *Worries are automatic, rapid, and often precede a powerful emotion*<br><br>Redirecting mind<br>• *Gently redirect mind like switching the channel to relaxing strategies*<br>• *Thought suppression doesn't work*<br>• *White bear activity*<br><br>Common thinking traps/cognitive distortions<br>• *Give one or two examples: e.g., personalization, black and white thinking etc.*<br><br>Relax the mind strategies<br>• *Relax the mind strategies help turn your attention to positive thoughts before bed and help you relax for sleep*<br>• *Gratitude practice: bring to mind things you are grateful for*<br>• *Savoring means taking the time to really enjoy how good something tastes/smells, or memories (use all five senses to make it vivid)*<br>• *Imagery is bringing to mind a relaxing scene*<br>• *Worry time is setting a specific time before bed to worry (at least 2 hours before) so the bed isn't associated with worry*<br>• *Problem-solving is listing your concerns and potential solutions to decrease worry*	

5- PROMOTING COMPLIANCE WITH CPAP MACHINE/ EXPOSURE THERAPY FOR CLAUSTROPHOBIC REACTIONS (7 treatment points)	Define sleep apnea, CPAP is 1st line treatment<br><br>Describe CPAP machine<br><br>Education on claustrophobic reactions with CPAP<br>● *Common and treatable*<br>● *Becoming more comfortable with CPAP while you are awake can help you learn to sleep easily with it*<br><br>Exposure therapy<br>● *Helps us face feared objects/situations with support*<br>● *By not escaping/avoiding, you increase control over your fear*<br>● *Only proceed to the next step in exposure if patient is comfortable with the previous step*	
6- NEGOTIATING SLEEP IN A COMPLICATED ENVIRONMENT (12 treatment points)	Environmental factors like noise and light make it difficult to sleep<br><br>Tips to improve environment<br>● *Earplugs, towels over windows, t-shirt under door frame, something over eyes*<br><br>SCALE technique to problem-solve<br>● *Specify the problem*<br>● *Consider all possible solutions*<br>● *Assess the best solution*<br>● *Lay out a plan*<br>● *Execute and evaluate the outcome*<br><br>Tips for interpersonal concerns (non-violent communication)<br>● *Observation: factual, non-judgmental, specific to incident, avoid always/never*<br>● *Feeling: "I" statements*<br>● *Need: without judgement*	

	• *Request: concrete, clear, specific*<br>• *Helpful to role-play*	
7- REDUCING NIGHTMARES<br>(5 treatment points)	Nightmares arise during REM (2nd half of night)<br><br>Imagery rehearsal<br>• *Imagery rehearsal can help reduce nightmares*<br>• *Think about a way to change the dream to be less distressing*<br>• *Rehearse multiple times with mental images*<br>• *No more than 2 dreams per week*	
Deviations from Gold Standard TranS-C material	*E.g., crystals help with your sleep*	

Table 2: Gold Standard Training Techniques

TRAINING TECHNIQUES	EXTENSIVENESS RATINGS (0-3)
**Agenda-setting**<br>*Trainer sets an agenda to set an organizational structure and facilitate effective time management.*<br><br>*E.g., The trainer may begin with a 'road map' to orient the trainees to the structure and timing of the training session and reference it throughout the training.*<br><br>Coding tips:<br>0 – No agenda set throughout training<br>3 – Agenda set at the beginning of training and referenced several times throughout	
**Behavioral Rehearsal (Role-play)**<br>*Trainer guides trainees through practicing effective use of a therapeutic skill or technique; trainees actively practice a skill playing the role of the therapist.*	

E.g., Trainer: "Okay, I'll pretend to be the patient —'but it's too hard! I can't get out of bed in the morning.'" [Trainee proceeds to play the clinician role and respond]<br><br>Coding tips:<br>Dialogue must be exchanged between "therapist" and "*patient*" with the trainee playing the therapist. An informal discussion about what to do with a *patient* does not count as role play. A formal role-play may rarely occur. Breakout discussion in which the trainer is not present does not count as role-play (see critical collaborative inquiry).<br><br>0 – no role plays<br>3 – role plays were used frequently throughout training (this is up to your judgement, based on length of training)<br><br>Please list the # of role plays here: ____	
**Trainer Modeling**<br>*Trainer models (i.e., enacts or demonstrates) a specific clinical skill or method of delivering a treatment component.*<br><br>*E.g., Trainer plays the role of the therapist to demonstrate proper delivery of TranS-C, such as leading attendees through an imagery exercise or an energy-generating experiment.*<br><br>Coding tips:<br>Small instances of modeling happen constantly throughout training ("I might explain this to a patient by saying…"); do not attempt to keep track of these. Only count "substantive" instances of modeling (hint: should be something like the trainer leading trainees through a Gratitude Practice). Playing a pre-recorded video of a role-play DOES count as modeling.<br><br>Examples: energy generating experiments (dance around!), gratitude practice, imagery, savoring (only if the trainer leads them through it – beyond just describing these things).<br><br>0 – modeling never used<br>3 – modeling used consistently throughout training<br><br>Please list the # of trainer modeling instances here: ____	

**Socratic Questioning**<br>*Trainer uses questions to promote critical thinking and active learning among trainees (e.g. probe assumptions, reasons, evidence), as opposed to providing 'answers'. These questions are designed to help trainees engage with TranS-C* ***content****.*<br><br>*E.g., "What may concern you about this sleep diary?" "How might you intervene?"*<br><br><u>Coding tips</u>:<br>You can tell it's Socratic Questioning if the trainer is asking a question of the participants *when the trainer clearly knows the answer*. Questions that would **NOT** count would be the trainer asking the trainees, "What would you like to work on in your sleep?" or "What is a wind-down routine you use?" – these will likely count as activities to promote active learning (below).<br><br>0 – Socratic questioning never used<br>3 – Socratic questioning used frequently throughout training	
**Positive Reinforcement / Praise**<br>*Trainer searches for, identifies, and labels positive aspects of trainees' work to reinforce strengths in their TranS-C delivery and participation in training.*<br><br>*E.g., "Great job, that's exactly right / you are on the right track." "Thank you for that comment, that's a great example."*<br><br><u>Coding tips</u>:<br>This will only happen when trainer is engaging with trainees.<br><br>0 – no positive reinforcement / praise used<br>3 – provided positive reinforcement in nearly every available opportunity	
**Discussion to Promote Self-reflection**	

Trainer encourages trainees to explore any thoughts, emotions, and actions that have arisen during the workshop with the goal of developing new understandings and appreciations. These questions should ***not*** *be about specific content (this is the key distinction from Socratic Questioning).*<br><br>*E.g., "What did you learn today about the patient perspective?" "How do you think your knowledge of TranS-C might impact your clinical practice more generally, and vice versa?" "What did you learn today that you didn't expect or that changed your perceptions about sleep?"*<br><br>Coding tips:<br>This will likely happen at the end of trainings or sections of modules (may not happen at all). These questions will not target a specific content item and will be general / open-ended.<br><br>0 – no self-reflection discussion attempted / occurred<br>3 – self-reflection discussion frequently attempted / occurred	
**Critical Collaborative Inquiry**<br>*Trainer promotes peer learning by encouraging peer contributions of experience, tips and skills. This might occur in breakout groups or trainer facilitating group discussion.*<br><br>*E.g., "What has been effective with your patients?" or "Does anyone have tips to share when patients don't complete homework?"*<br><br>Coding tips:<br>Trainees should be discussing and learning from each other, rather than from the trainer. Trainer should directly facilitate this (e.g., breakout group) or in group discussion.<br><br>0 – no critical collaborative inquiry attempted / occurred<br>3 – critical collaborative inquiry frequently attempted / occurred<br><br>Please list # of breakout groups: ______	
**Other Activities to Promote Active Learning**<br>*Trainer uses activities to promote engagement with TranS-C material.*	

E.g., Trainee engages trainee participation with polls, filling out patient worksheets, calculating sleep variables, or facilitating group discussion. *<u>Do not count showing videos</u>.*<br><br><u>Coding tips:</u><br>*Be careful not to double-count role-plays or critical collaborative inquiry here.* If it is another instance in which the trainer is engaging trainees, it is likely an activity to promote active learning.<br><br>0 – no activities attempted / occurred<br>3 – activities frequently attempted / occurred	
**Trainee Response and Engagement**<br>*Trainees engage in discussion and are responsive to trainers' attempts to promote active learning.*<br><br>*E.g., Trainees respond to polls, trainer questions and ask questions of their own.*<br><br>0 – no trainee engagement<br>3 – trainees highly engaged throughout training	

Note Recording Mishaps here:

E.g., The recording started partway through teaching module 1. An agenda for the training was referenced later and it was clear that it was set at the beginning as well.
